# Supplementary figures and images for: Deconvolving sequence features that discriminate between overlapping regulatory annotations
Source: PLoS Comput Biol. 2017 Oct 19;13(10):e1005795. doi: 10.1371/journal.pcbi.1005795 (PMC5663517; doi:10.1371/journal.pcbi.1005795)

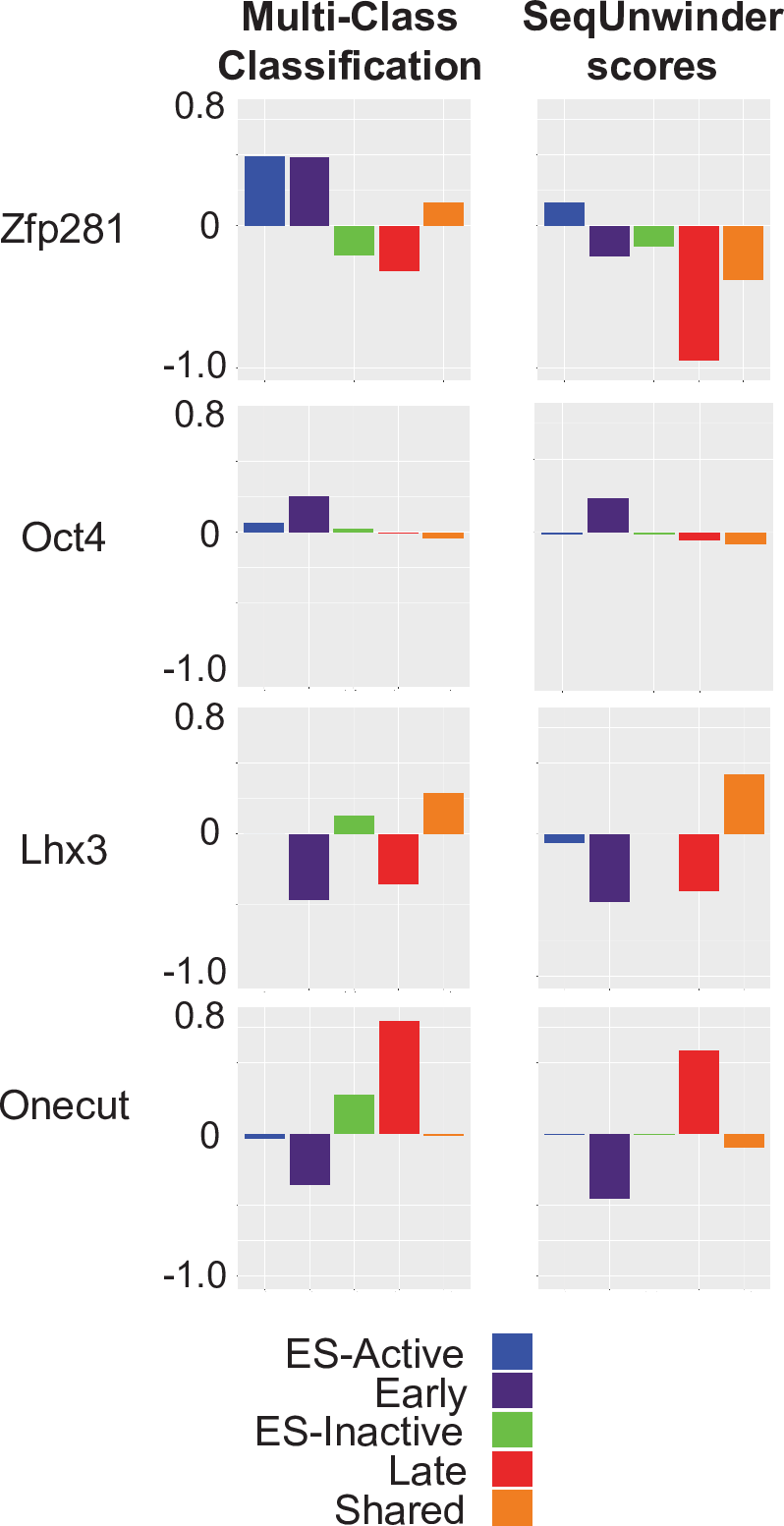

Supplement: S1 Fig — SeqUnwinder and MCC model scores of de novo discovered Zfp281-like, Oct4-like, Lhx3-like and Onecut-like motifs in various Isl1/Lhx3 site categories. Positive scores denote that the motif is positively discriminative of the given label (relative to the other labels), and vice versa for negative scores. (TIF) [file pcbi.1005795.s001.tif]

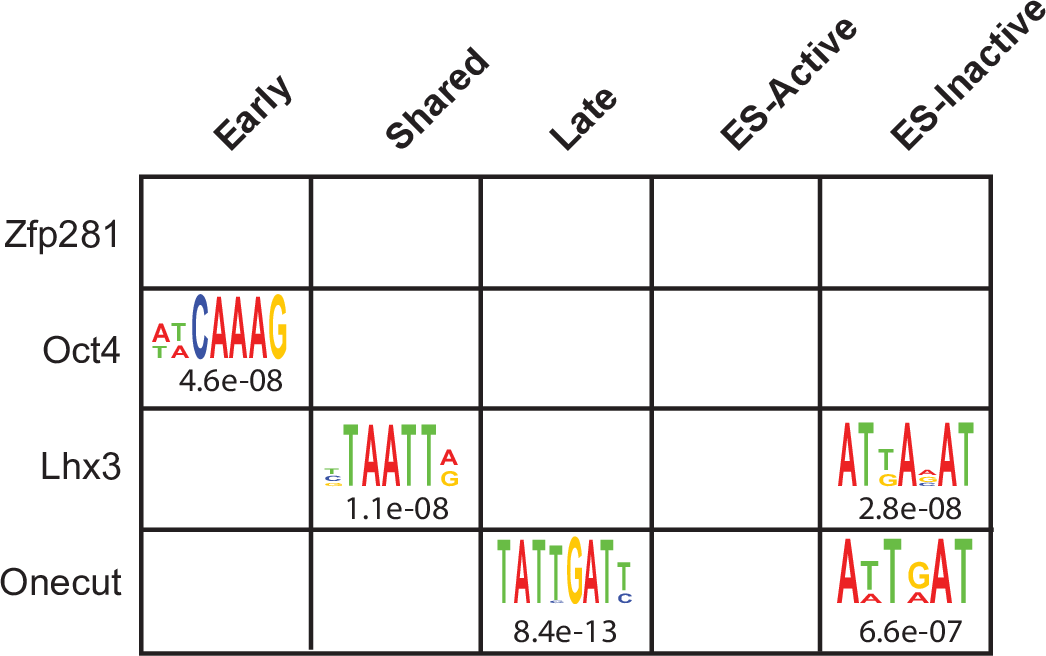

Supplement: S2 Fig — This figure summarizes whether DREME finds Zfp281-like, Oct4-like, Lhx3-like and Onecut-like motifs in analyses that aim to discriminate a given label’s sites against sites from all other labels. Displayed motifs match the relevant TF’s motif in the cis-bp database according to STAMP analysis (values listed under the motifs correspond to STAMP E-values). (TIF) [file pcbi.1005795.s002.tif]

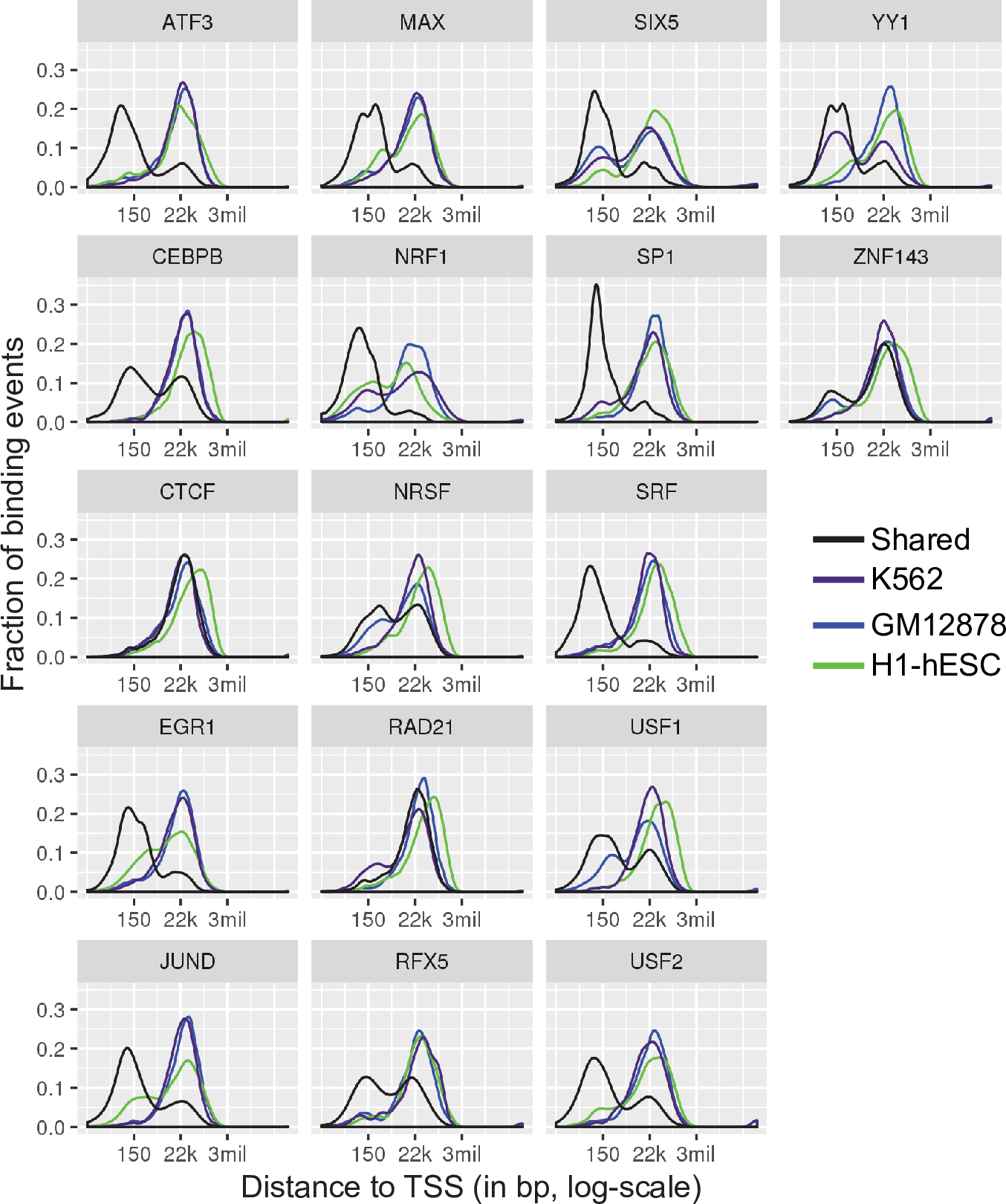

Supplement: S3 Fig — The distributions show the distances of TF binding events from annotated mRNA TSS for all 17 examined ENCODE TFs, stratified based on “shared” (black) or “cell line-specific” labels (purple = K562, blue = GM12878, green = H1-hESC). The X-axis represents the distance in base pairs plotted according to a log-scale (natural logarithm). (TIF) [file pcbi.1005795.s003.tif]

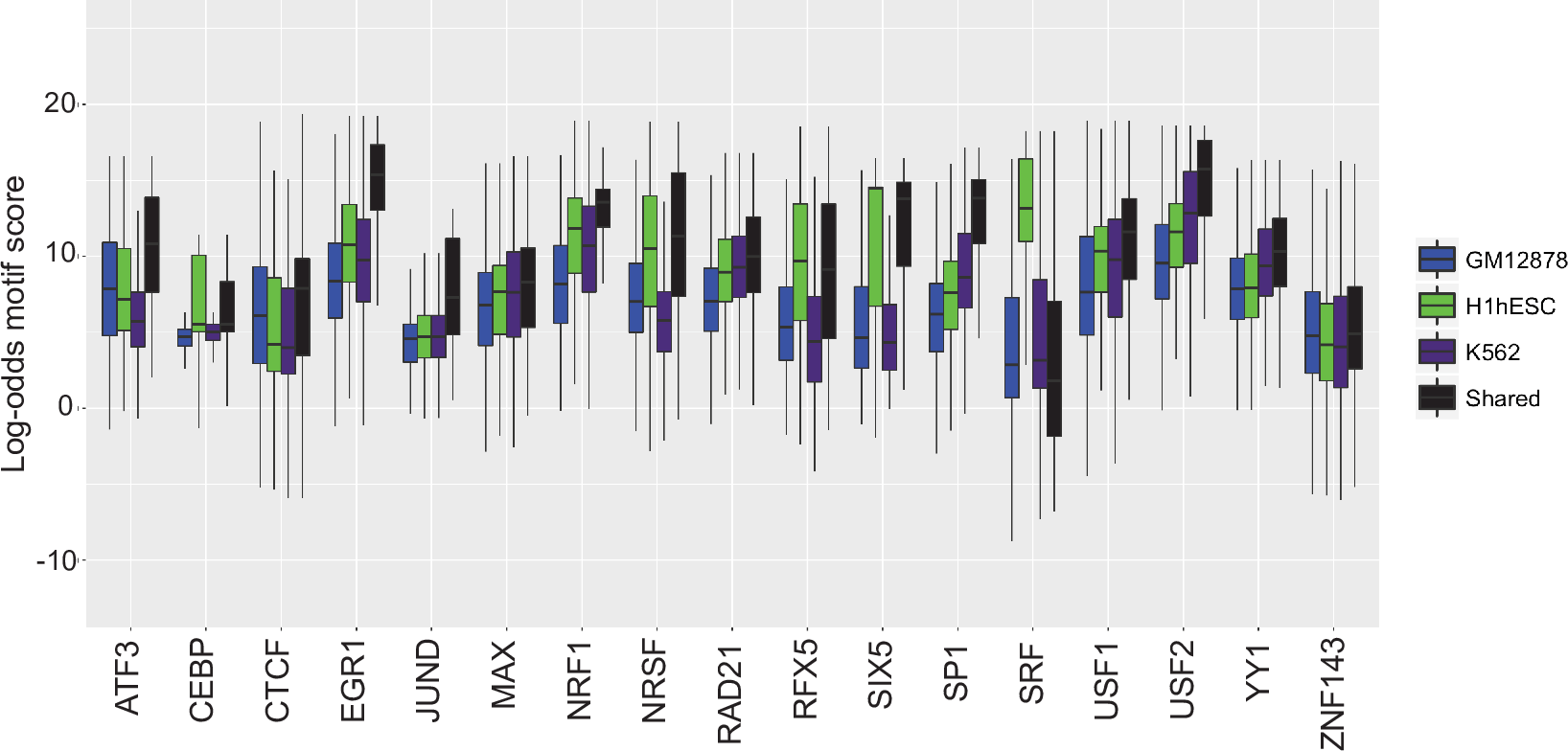

Supplement: S4 Fig — Log-odds score distributions summarizing highest-scoring instances of SeqUnwinder’s de novo discovered cognate motifs at “shared”, “K562”, “GM12878” and “H1-hESC” labeled binding events. (TIF) [file pcbi.1005795.s004.tif]

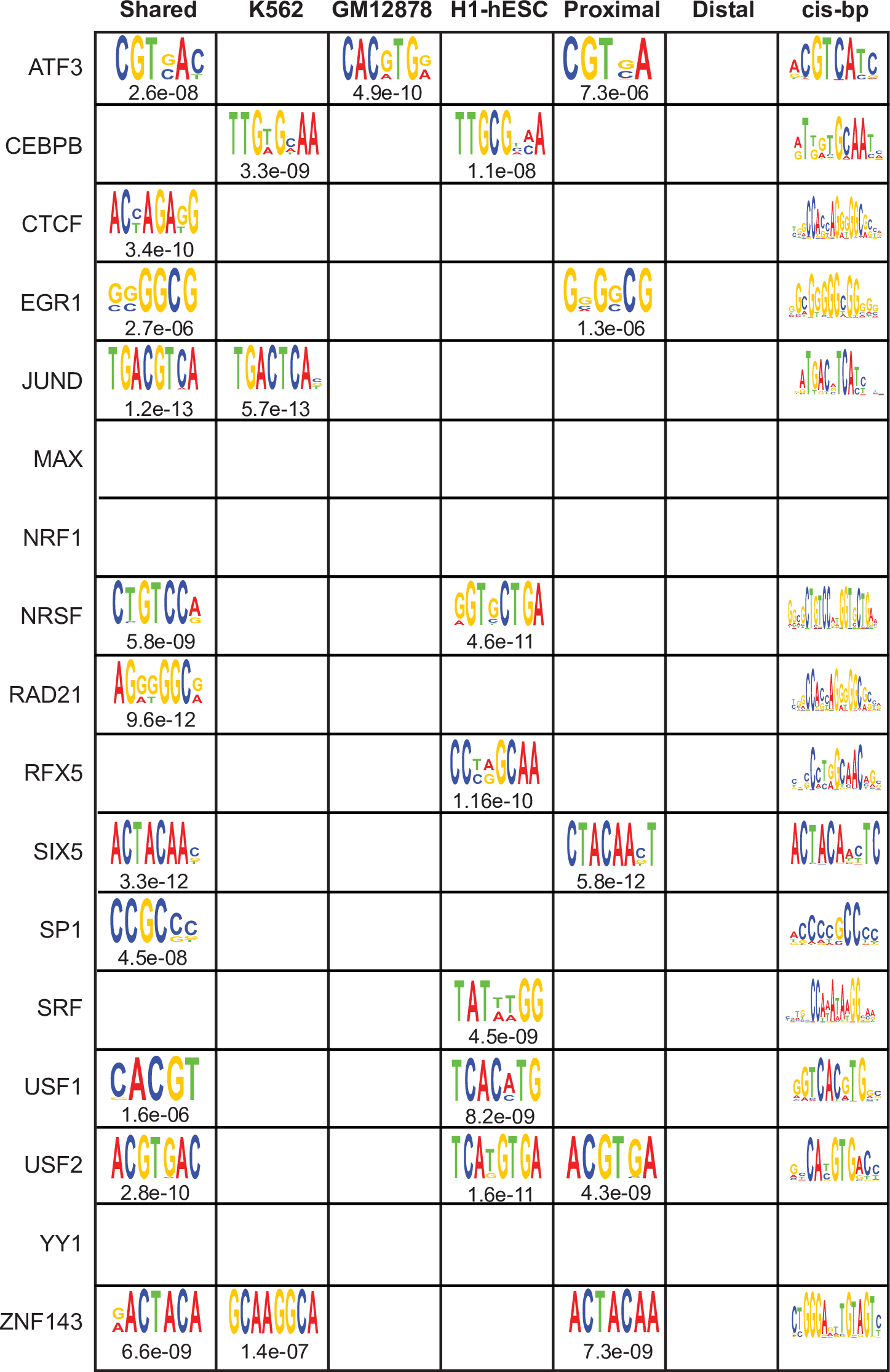

Supplement: S5 Fig — This figure summarizes whether DREME finds cognate motifs in analyses that aim to discriminate a given label’s sites against sites from all other labels. Displayed motifs match the relevant cognate motifs in the cis-bp database according to STAMP analysis (values listed under the motifs correspond to STAMP E-values). (TIF) [file pcbi.1005795.s005.tif]
