# Supplementary material for: Deconvolving sequence features that discriminate between overlapping regulatory annotations
Source: PLoS Comput Biol. 2017 Oct 19;13(10):e1005795. doi: 10.1371/journal.pcbi.1005795 (PMC5663517; doi:10.1371/journal.pcbi.1005795)
Supplement: S1 Table — Area under receiver operating characteristic curve (auROC) values describing the classification performance of SeqUnwinder for each Isl1/Lhx3 subclass. Classification performance is determined using 3-fold cross-validation. (DOCX) [file pcbi.1005795.s006.docx]

| **Subclass** | **auROC** |
| --- | --- |
| ES-Active & Shared | 0.692 |
| ES-Inactive & Shared | 0.684 |
| ES-Active & Early | 0.778 |
| ES-Inactive & Early | 0.736 |
| ES-Active & Late | 0.68 |
| ES-Inactive & Late | 0.772 |
| *Average* | *0.723* |

**S1 Table. Performance of SeqUnwinder in classifying each Isl1/Lhx3 binding site subclass.**

Area under receiver operating characteristic curve (auROC) values describing the classification performance of SeqUnwinder for each Isl1/Lhx3 subclass. Classification performance is determined using 3-fold cross-validation.
