# Supplementary material for: Deconvolving sequence features that discriminate between overlapping regulatory annotations
Source: PLoS Comput Biol. 2017 Oct 19;13(10):e1005795. doi: 10.1371/journal.pcbi.1005795 (PMC5663517; doi:10.1371/journal.pcbi.1005795)
Supplement: S2 Table — Area under receiver operating characteristic curve (auROC) values describing the classification performance of SeqUnwinder for each subclass of binding sites. Classification performance is determined using 3-fold cross-validation. (DOCX) [file pcbi.1005795.s007.docx]

| **Dataset** | **Shared and Proximal** | **Shared and Distal** | **K562 and Proximal** | **K562 and Distal** | **GM12878 and Proximal** | **GM12878 and Distal** | **H1hESC and Distal** | **H1hESC and Proximal** | ***Average auROC*** |
| --- | --- | --- | --- | --- | --- | --- | --- | --- | --- |
| ATF3 | 0.913 | 0.729 | 0.759 | 0.849 | 0.660 | 0.801 | 0.728 | 0.770 | *0.776* |
| CEBPB | 0.890 | 0.744 | 0.768 | 0.835 | 0.770 | 0.814 | 0.777 | 0.806 | *0.801* |
| CTCF | 0.688 | 0.674 | 0.680 | 0.699 | 0.615 | 0.739 | 0.768 | 0.758 | *0.703* |
| EGR1 | 0.896 | 0.775 | 0.772 | 0.859 | 0.751 | 0.862 | 0.846 | 0.811 | *0.822* |
| JUND | 0.915 | 0.683 | 0.791 | 0.860 | 0.755 | 0.867 | 0.884 | 0.816 | *0.821* |
| MAX | 0.867 | 0.724 | 0.692 | 0.825 | 0.700 | 0.832 | 0.818 | 0.785 | *0.780* |
| NRF1 | 0.801 | 0.645 | 0.623 | 0.703 | NA | NA | 0.667 | 0.613 | *0.675* |
| NRSF | 0.830 | 0.784 | 0.844 | 0.891 | 0.839 | 0.839 | 0.762 | 0.810 | *0.824* |
| RAD21 | 0.757 | 0.748 | 0.857 | 0.746 | 0.775 | 0.846 | 0.797 | 0.776 | *0.788* |
| RFX5 | 0.809 | 0.521 | 0.666 | 0.823 | 0.658 | 0.817 | 0.677 | 0.783 | *0.719* |
| SIX5 | 0.866 | 0.807 | NA | NA | 0.683 | 0.764 | 0.595 | 0.791 | *0.751* |
| SP1 | 0.910 | 0.799 | 0.774 | 0.864 | 0.785 | 0.889 | 0.802 | 0.861 | *0.836* |
| SRF | 0.910 | 0.649 | 0.675 | 0.789 | 0.775 | 0.811 | 0.743 | 0.848 | *0.775* |
| USF1 | 0.893 | 0.788 | 0.752 | 0.830 | 0.870 | 0.837 | 0.749 | 0.850 | *0.821* |
| USF2 | 0.909 | 0.784 | 0.614 | 0.775 | 0.745 | 0.824 | 0.765 | 0.731 | *0.768* |
| YY1 | 0.866 | 0.741 | 0.864 | 0.808 | 0.797 | 0.87 | 0.802 | 0.810 | *0.819* |
| ZNF143 | 0.845 | 0.796 | 0.654 | 0.774 | 0.777 | 0.814 | 0.807 | 0.765 | *0.779* |

**S2 Table. Performance of SeqUnwinder in classifying subclasses of binding sites for seventeen ENCODE TFs.**

Area under receiver operating characteristic curve (auROC) values describing the classification performance of SeqUnwinder for each subclass of binding sites. Classification performance is determined using 3-fold cross-validation.
