# Supplementary material for: Deconvolving sequence features that discriminate between overlapping regulatory annotations
Source: PLoS Comput Biol. 2017 Oct 19;13(10):e1005795. doi: 10.1371/journal.pcbi.1005795 (PMC5663517; doi:10.1371/journal.pcbi.1005795)
Supplement: S3 Table — Area under receiver operating characteristic curve (auROC) values describing the classification performance of SeqUnwinder for each subclass of DHS sites. Classification performance is determined using 3-fold cross-validation. (DOCX) [file pcbi.1005795.s008.docx]

| **Subclass** | **auROC** |
| --- | --- |
| H1hESC-only | 0.788 |
| HeLa-S3-only | 0.809 |
| HeLa-S3and HUVEC | 0.830 |
| GM12878 and H1hESC | 0.766 |
| GM12878-only | 0.810 |
| HUVEC -only | 0.831 |
| K562-only | 0.806 |
| GM12878 and K562 | 0.774 |
| Shared w/o K562 | 0.813 |
| HepG2-only | 0.773 |
| GM12878 and HUVEC | 0.750 |
| H1hESC and HeLa-S3 | 0.757 |
| Shared | 0.790 |
| K562 and HepG2 | 0.737 |
| GM12878 and HeLa-S3 | 0.772 |
| GM12878 and HepG2 | 0.773 |
| Shared w/o HepG2 | 0.785 |
| HepG2 and HUVEC | 0.744 |
| K562 and HUVEC | 0.772 |
| H1hESC and HepG2 | 0.827 |
| Shared w/o GM12878 | 0.791 |
| Shared w/o HeLa-S3 | 0.873 |
| Shared w/o H1hESC | 0.772 |
| HeLa-S3and HepG2 | 0.741 |
| K562 and HeLa-S3 | 0.758 |
| K562 and H1hESC | 0.767 |
| H1hESC and HUVEC | 0.721 |
| Shared w/o HUVEC | 0.838 |

**S3 Table. Performance of SeqUnwinder model in classifying subclasses of DHS sites in 6 different ENCODE cell-lines**.

Area under receiver operating characteristic curve (auROC) values describing the classification performance of SeqUnwinder for each subclass of DHS sites. Classification performance is determined using 3-fold cross-validation.
